# Association between Body Roundness Index and infertility risk in Han Chinese women: a retrospective observational study

**DOI:** 10.3389/fmed.2026.1714709

**Published:** 2026-02-11

**Authors:** Nizhou Liu, Xingyu Sun, Lijuan He

**Affiliations:** 1 Department of Reproductive Medicine, The Affiliated Hospital, Southwest Medical University, Luzhou, Sichuan, China; 2 Department of Gynecology, The Affiliated Traditional Chinese Medicine Hospital, Southwest Medical University, Luzhou, Sichuan, China; 3 Department of Health Management Center, The Affiliated Hospital, Southwest Medical University, Luzhou, Sichuan, China

**Keywords:** Body Roundness Index, central adiposity, Han Chinese women, infertility, retrospective observational study

## Abstract

**Background:**

Infertility is a major global health concern and is increasingly recognized to be influenced by metabolic and anthropometric factors. The Body Roundness Index (BRI), a waist- and height-based indicator of central adiposity, has been associated with cardiometabolic outcomes. Although emerging studies have examined the relationship between BRI and infertility, evidence remains limited across populations, particularly regarding non-linear associations and effect modification.

**Objective:**

This study aimed to examine the association between BRI and infertility risk in Han Chinese women and to explore potential non-linear and subgroup-specific patterns.

**Methods:**

We conducted a retrospective observational study including 425 Han Chinese women aged 20–44 years. BRI was calculated from measured waist circumference and height. Infertility was defined as failure to conceive after 12 months of unprotected intercourse. Multivariable logistic regression was used to evaluate the association between BRI and infertility after adjustment for selected confounders, including age, Body Mass Index, physical activity, and Polycystic Ovary Syndrome. Restricted cubic spline functions within logistic regression were applied to assess potential non-linear relationships, and subgroup analyses were performed to evaluate effect modification.

**Results:**

Women with infertility had significantly higher BRI values than those without infertility (8.5 ± 2.0 vs. 7.1 ± 1.5, *p* < 0.001). After multivariable adjustment, each unit increase in BRI was associated with higher odds of infertility (adjusted OR = 1.38, 95% CI: 1.22–1.56). Restricted cubic spline analysis demonstrated a non-linear dose-response relationship, with infertility risk increasing more steeply at BRI levels above approximately 8.0. Stronger associations were observed among women aged 35–44 years, those with higher BMI, sedentary lifestyles, Polycystic Ovary Syndrome, or lower income (*p* for interaction <0.05).

**Conclusion:**

Higher BRI is associated with increased infertility risk among Han Chinese women, with a non-linear pattern that becomes more pronounced at higher levels of central adiposity. These findings suggest that BRI may serve as a useful anthropometric marker for infertility risk stratification, although confirmation in larger prospective studies with more comprehensive covariate assessment is warranted.

## Introduction

Infertility is a clinically important reproductive outcome that is closely intertwined with endocrine and metabolic health, and accumulating evidence suggests that central (visceral) adiposity may be more relevant to reproductive dysfunction than overall body mass alone (1). In the present study, we focused on Han Chinese women to enhance population homogeneity and internal validity, as ethnic differences in body fat distribution and metabolic susceptibility may modify the association between central adiposity and reproductive outcomes; moreover, evidence specific to Han Chinese women remains relatively limited. This condition not only poses substantial emotional and social challenges but also has significant public health implications due to its increasing prevalence. Among the numerous factors influencing infertility, abdominal obesity has emerged as a critical determinant, mediated by hormonal dysregulation, chronic inflammation, and metabolic disturbances (2).

The traditional metric for assessing obesity, the Body Mass Index (BMI), has been widely used in clinical and research settings. However, BMI does not adequately capture regional fat distribution, and central (visceral) adiposity appears to be more closely linked to endocrine and metabolic disturbances relevant to reproductive function than overall body mass alone (3). Because visceral fat is metabolically active and more strongly linked to insulin resistance, hyperandrogenism, and inflammatory signaling, indices emphasizing abdominal adiposity may better capture the pathways most relevant to fecundability than BMI. Recent attention has turned to alternative anthropometric indices, such as the BRI, which incorporates both height and waist circumference to provide a more precise measure of abdominal fat distribution (4). BRI has demonstrated strong correlations with cardiometabolic conditions, such as diabetes and cardiovascular disease, and is considered superior to BMI in predicting central obesity-related health risks (5). With these advances, investigations of the relationship between BRI and infertility have commenced in women and in men (6–8); however, existing studies are largely cross-sectional and population-specific, and evidence on dose–response shape (non-linearity) and effect modification in Han Chinese women remains scarce (9).

Abdominal obesity is hypothesized to contribute to infertility through mechanisms involving insulin resistance, altered sex hormone levels, and impaired ovarian function (10). Mechanistically, visceral adiposity is metabolically active and may promote insulin resistance and hyperinsulinemia, reduce SHBG, and exacerbate ovarian androgen excess, thereby impairing folliculogenesis and ovulation. In parallel, adipokine dysregulation and low-grade inflammation related to visceral fat may perturb gonadotropin signaling and endometrial receptivity, providing biological plausibility for BRI as a marker of infertility risk (11, 12). These processes are particularly relevant in conditions such as Polycystic Ovary Syndrome (PCOS), where abdominal fat accumulation exacerbates reproductive dysfunction (13). Understanding the role of BRI in predicting infertility could offer valuable insights into its utility as a clinical tool for risk stratification and early intervention. Notably, central adiposity may also adversely affect male fertility via impaired semen quality (e.g., reduced concentration and motility, increased DNA fragmentation), hormonal disruption, and heightened systemic inflammation and oxidative stress, underscoring the couple-level nature of infertility (14).

In this study, we aimed to investigate the association between BRI and infertility risk in Han Chinese women of reproductive age. Specifically, we sought to examine the independent association between BRI and infertility, to characterize potential non-linear patterns using restricted cubic spline modeling, and to evaluate effect modification by key demographic, anthropometric, and lifestyle factors, including age, BMI, physical activity, and socioeconomic status. By focusing on a relatively understudied population and incorporating non-linear and interaction analyses, this study aims to extend existing evidence on the relevance of advanced anthropometric indices for reproductive health assessment.

## Methods

### Study design and population

**Figure 1:**
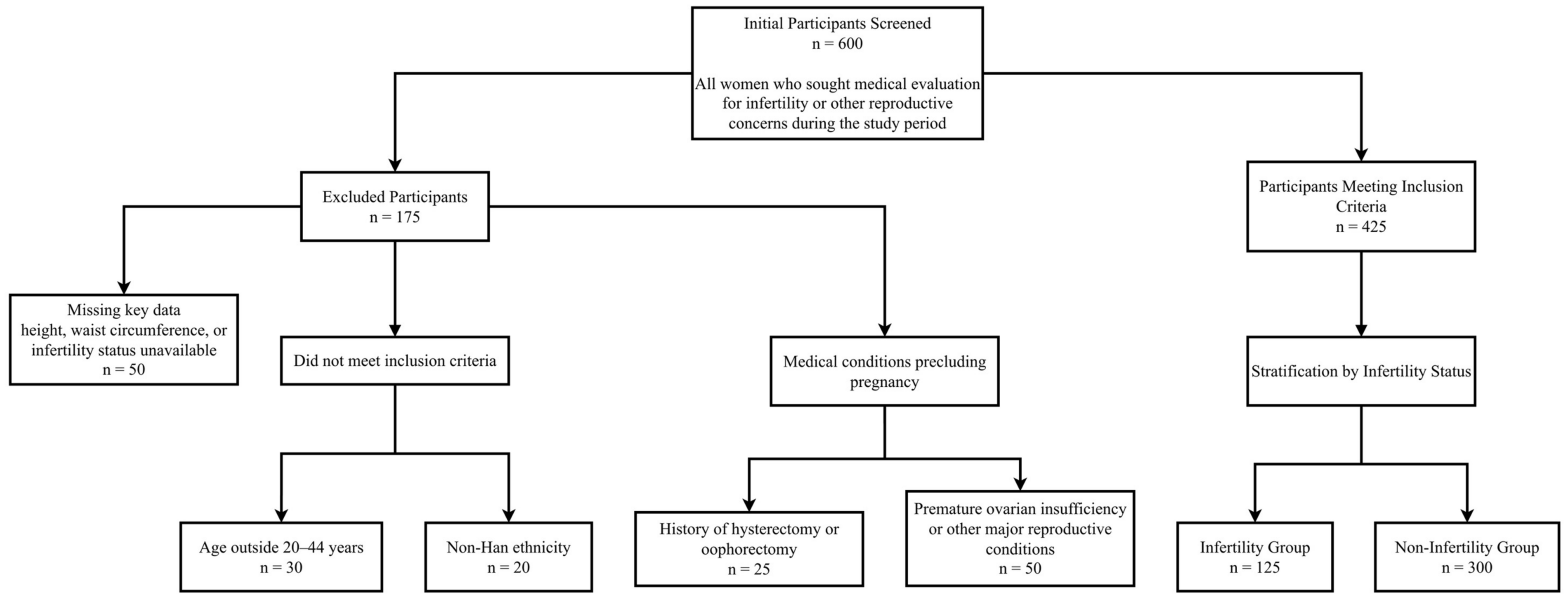
Flowchart of participant enrollment and stratification in the study.

This retrospective observational study was conducted at a tertiary hospital. We screened all women who sought medical evaluation for infertility or other reproductive concerns between January 2020 and December 2023 (*n* = 600). Eligible participants were Han Chinese women aged 20–44 years with available anthropometric measurements (height and waist circumference) and sufficient information to determine infertility status. Women were excluded if they had conditions precluding pregnancy (e.g., hysterectomy, bilateral oophorectomy, or premature ovarian insufficiency) or if key variables required for BRI calculation or outcome ascertainment were missing. Of the 600 women screened, 175 were excluded for the following reasons: missing key data (*n* = 50), not meeting the predefined age range of 20–44 years (*n* = 30), non-Han ethnicity (*n* = 20), and medical conditions precluding pregnancy (*n* = 75). The final analytic sample included 425 women, of whom 125 were classified as infertile and 300 as non-infertile (Figure 1).

### Exposure variable

Waist circumference was measured at the midpoint between the lower costal margin and the iliac crest, and height was measured without shoes, both in centimeters. BRI was calculated according to the original geometric body-shape model proposed by Thomas et al. (15). Specifically, waist circumference (WC) and height were measured in centimeters, and the waist-to-height ratio was computed as WHtR = WC/Height. Eccentricity was then derived as *e* = √[1 − (WHtR/*π*)^2^], and BRI was calculated as BRI = 364.2–365.5 × *e*. All BRI values were computed directly from the measured anthropometric data using custom code in R (version 4.1.0), rather than an online BRI calculator, to ensure full reproducibility. BRI was analyzed as a continuous variable and categorized into quartiles based on the distribution in the study population: Q1 (≤6.5), Q2 (6.6–7.5), Q3 (7.6–8.5), and Q4 (>8.5). Information on male-partner factors (e.g., semen parameters) and other couple-level determinants was not uniformly available in the medical records and therefore could not be included in the adjusted models.

### Outcome variable

The primary outcome was infertility, defined as the inability to conceive after 12 months of regular, unprotected intercourse, as self-reported or clinically diagnosed by reproductive specialists.

### Covariates

Covariates included age (years), BMI (kg/m^2^), physical activity (regular vs. sedentary), PCOS diagnosis (yes vs. no), history of pelvic infection (yes vs. no), irregular menstruation (yes vs. no), hormonal treatment history (yes vs. no), and monthly income (<5,000 CNY vs. ≥5,000 CNY). Information on metabolic comorbidities (e.g., physician-diagnosed diabetes, dyslipidemia, and hypertension) was not consistently recorded for all participants in the retrospective medical records and therefore was not included in the multivariable adjustment.

### Statistical analysis

Baseline characteristics were summarized as mean (SD) for continuous variables and as frequencies (percentages) for categorical variables. Group differences between infertility and non-infertility groups were assessed using independent *t*-tests for continuous variables and chi-square tests for categorical variables.

Multivariate logistic regression was performed to estimate odds ratios (ORs) and 95% confidence intervals (CIs) for the association between BRI and infertility, adjusting for potential confounders. Restricted cubic spline (RCS) functions were applied to model the potential non-linear association between BRI and infertility risk within the multivariable logistic regression framework. We used 4 knots placed at the 5th, 35th, 65th, and 95th percentiles of the BRI distribution, with BRI = 6.5 as the reference value. A likelihood ratio test comparing the model with only the linear term to the model including the spline terms was used to formally assess non-linearity (*p* for non-linearity). Subgroup analyses were conducted to evaluate effect modification by age, BMI, physical activity, PCOS diagnosis, and income, and interaction terms were tested for statistical significance. All analyses were performed using R software (version 4.1.0). A two-tailed *p*-value of <0.05 was considered statistically significant. Multicollinearity was assessed using variance inflation factors, and no evidence of problematic multicollinearity was detected.

## Results

### Baseline characteristics of the study population

Table 1 presents the baseline characteristics of the study population, stratified by infertility status. The mean age of participants in the infertility group was significantly higher than that of the non-infertility group (33.9 ± 6.8 years vs. 30.9 ± 5.9 years, *p* < 0.001), suggesting age may play a role in infertility risk. Notably, the mean BRI was substantially elevated in the infertility group compared to the non-infertility group (8.5 ± 2.0 vs. 7.1 ± 1.5, *p* < 0.001), highlighting a potential association between abdominal adiposity and infertility. Similarly, the infertility group had higher mean BMI (27.2 ± 4.8 vs. 24.3 ± 4.0, *p* < 0.001) and WC (86.8 ± 12.2 cm vs. 79.3 ± 10.8 cm, *p* < 0.001), further emphasizing the role of body composition in reproductive health. Educational attainment differed significantly between groups (*p* = 0.021), with a higher proportion of infertility cases having attained a college or higher education level (48.0% vs. 36.7%). Lifestyle factors such as regular physical activity were notably less common in the infertility group (56.0% vs. 75.0%, *p* < 0.001), while smoking and alcohol consumption did not differ significantly between groups. Regarding reproductive history, the infertility group demonstrated a markedly higher prevalence of PCOS (29.6% vs. 11.7%, *p* < 0.001), irregular menstruation (40.0% vs. 18.3%, *p* < 0.001), and prior pelvic infection (16.0% vs. 6.7%, *p* = 0.002). Additionally, hormonal treatment use was significantly more frequent in the infertility group (40.0% vs. 15.0%, *p* < 0.001). Lastly, a lower proportion of participants in the infertility group reported monthly incomes ≥5,000 CNY (44.0% vs. 61.7%, *p* < 0.001), suggesting potential socioeconomic disparities.

**Table 1:** Baseline characteristics of the study population.

Characteristics	Overall (*n* = 425)	Non-infertility (*n* = 300)	Infertility (*n* = 125)	*p*-value
Demographics				
Age (years), mean (SD)	31.8 (6.4)	30.9 (5.9)	33.9 (6.8)	<0.001**
BRI, mean (SD)	7.5 (1.8)	7.1 (1.5)	8.5 (2.0)	<0.001**
BMI (kg/m^2^), mean (SD)	25.2 (4.5)	24.3 (4.0)	27.2 (4.8)	<0.001**
Waist circumference (cm), mean (SD)	81.5 (11.5)	79.3 (10.8)	86.8 (12.2)	<0.001**
Education, *n* (%)				0.021*
Less than high school	50 (11.8%)	35 (11.7%)	15 (12.0%)	
High school	205 (48.2%)	155 (51.7%)	50 (40.0%)	
College or higher	170 (40.0%)	110 (36.7%)	60 (48.0%)	
Lifestyle factors				
Smoking, *n* (%)	52 (12.2%)	35 (11.7%)	17 (13.6%)	0.520
Alcohol consumption, *n* (%)	190 (44.7%)	140 (46.7%)	50 (40.0%)	0.180
Regular physical activity, *n* (%)	295 (69.4%)	225 (75.0%)	70 (56.0%)	<0.001**
Reproductive history				
PCOS diagnosis, *n* (%)	72 (16.9%)	35 (11.7%)	37 (29.6%)	<0.001**
Irregular menstruation, *n* (%)	105 (24.7%)	55 (18.3%)	50 (40.0%)	<0.001**
Pelvic infection history, *n* (%)	40 (9.4%)	20 (6.7%)	20 (16.0%)	0.002**
Hormonal treatment, *n* (%)	95 (22.4%)	45 (15.0%)	50 (40.0%)	<0.001**
Economic factors				
Monthly income ≥5,000 CNY, *n* (%)	240 (56.5%)	185 (61.7%)	55 (44.0%)	<0.001**

Values are presented as mean (SD) for continuous variables and *n* (%) for categorical variables. BRI, Body Roundness Index; BMI, Body Mass Index; PCOS, Polycystic Ovary Syndrome; CNY, Chinese Yuan. *p*-values were calculated using *t*-tests for continuous variables and chi-square tests for categorical variables to compare differences between the infertility and non-infertility groups. Statistical significance was defined as *p* < 0.05.

*Indicates *p* < 0.05; **Indicates *p* < 0.01. All *p*-values are two-sided.

### Multivariate logistic regression analysis of factors associated with infertility

Table 2 presents the results of the multivariate logistic regression analysis examining factors associated with infertility. Body Roundness Index emerged as a significant predictor, with each unit increase in BRI associated with a 38% higher odds of infertility after adjusting for confounders (adjusted OR: 1.38, 95% CI: 1.22–1.56, *p* < 0.001). When BRI was analyzed as a categorical variable, a dose–response relationship was observed: participants in the highest quartile of BRI (Q4, >8.5) had more than three times the odds of infertility compared to those in the lowest quartile (Q1, ≤6.5; adjusted OR: 3.12, 95% CI: 2.11–4.61, *p* < 0.001). BMI was also significantly associated with infertility, with a modest but independent increase in risk per unit increase (adjusted OR: 1.12, 95% CI: 1.05–1.20, *p* < 0.001). Age similarly showed a positive association, with older women experiencing a greater likelihood of infertility (adjusted OR: 1.06, 95% CI: 1.02–1.10, *p* = 0.002). Reproductive history variables demonstrated strong associations. Women with PCOS had nearly three times the odds of infertility compared to those without (adjusted OR: 2.85, 95% CI: 1.84–4.40, *p* < 0.001). Similarly, irregular menstruation (adjusted OR: 2.57, 95% CI: 1.73–3.82, *p* < 0.001) and a history of pelvic infection (adjusted OR: 2.40, 95% CI: 1.26–4.58, *p* = 0.008) were significant risk factors. Socioeconomic and lifestyle factors were also relevant. Higher monthly income (≥5,000 CNY) was associated with reduced odds of infertility (adjusted OR: 0.65, 95% CI: 0.44–0.96, *p* = 0.029), suggesting a protective effect of better economic status. Regular physical activity was similarly protective, reducing the odds of infertility by 40% (adjusted OR: 0.60, 95% CI: 0.41–0.88, *p* = 0.009).

**Table 2:** Multivariate logistic regression analysis of factors associated with infertility.

Variable	Crude OR (95% CI)	Adjusted OR (95% CI)	*p*-value
Body Roundness Index (BRI)			
Continuous (per unit increase)	1.45 (1.30–1.62)	1.38 (1.22–1.56)	<0.001**
Quartiles			
Q1 (≤6.5, reference)	1.00 (reference)	1.00 (reference)	–
Q2 (6.6–7.5)	1.32 (0.92–1.88)	1.25 (0.88–1.76)	0.210
Q3 (7.6–8.5)	2.10 (1.48–2.97)	1.98 (1.37–2.87)	<0.001**
Q4 (>8.5)	3.56 (2.47–5.13)	3.12 (2.11–4.61)	<0.001**
BMI (per unit increase)	1.18 (1.10–1.26)	1.12 (1.05–1.20)	<0.001**
Age (years)	1.07 (1.03–1.11)	1.06 (1.02–1.10)	0.002**
PCOS diagnosis (yes vs. no)	3.12 (2.05–4.75)	2.85 (1.84–4.40)	<0.001**
Irregular menstruation (yes vs. no)	2.80 (1.91–4.11)	2.57 (1.73–3.82)	<0.001**
Pelvic infection history	2.74 (1.46–5.13)	2.40 (1.26–4.58)	0.008**
Monthly income ≥5,000 CNY	0.58 (0.41–0.83)	0.65 (0.44–0.96)	0.029*
Regular physical activity	0.52 (0.36–0.74)	0.60 (0.41–0.88)	0.009**

The table presents the results of logistic regression analysis. Crude ORs were calculated without adjusting for confounders, while adjusted ORs (AORs) were adjusted for age, BMI, PCOS diagnosis, physical activity, monthly income, and other relevant covariates. BRI, Body Roundness Index; BMI, Body Mass Index; PCOS, Polycystic Ovary Syndrome. Statistical significance was defined as *p* < 0.05. 95% CI, 95% confidence interval.

*Indicates *p* < 0.05; **Indicates *p* < 0.01. All *p*-values are two-sided.

### Restricted cubic spline (RCS) analysis of the association between BRI and infertility risk

**Figure 2:**
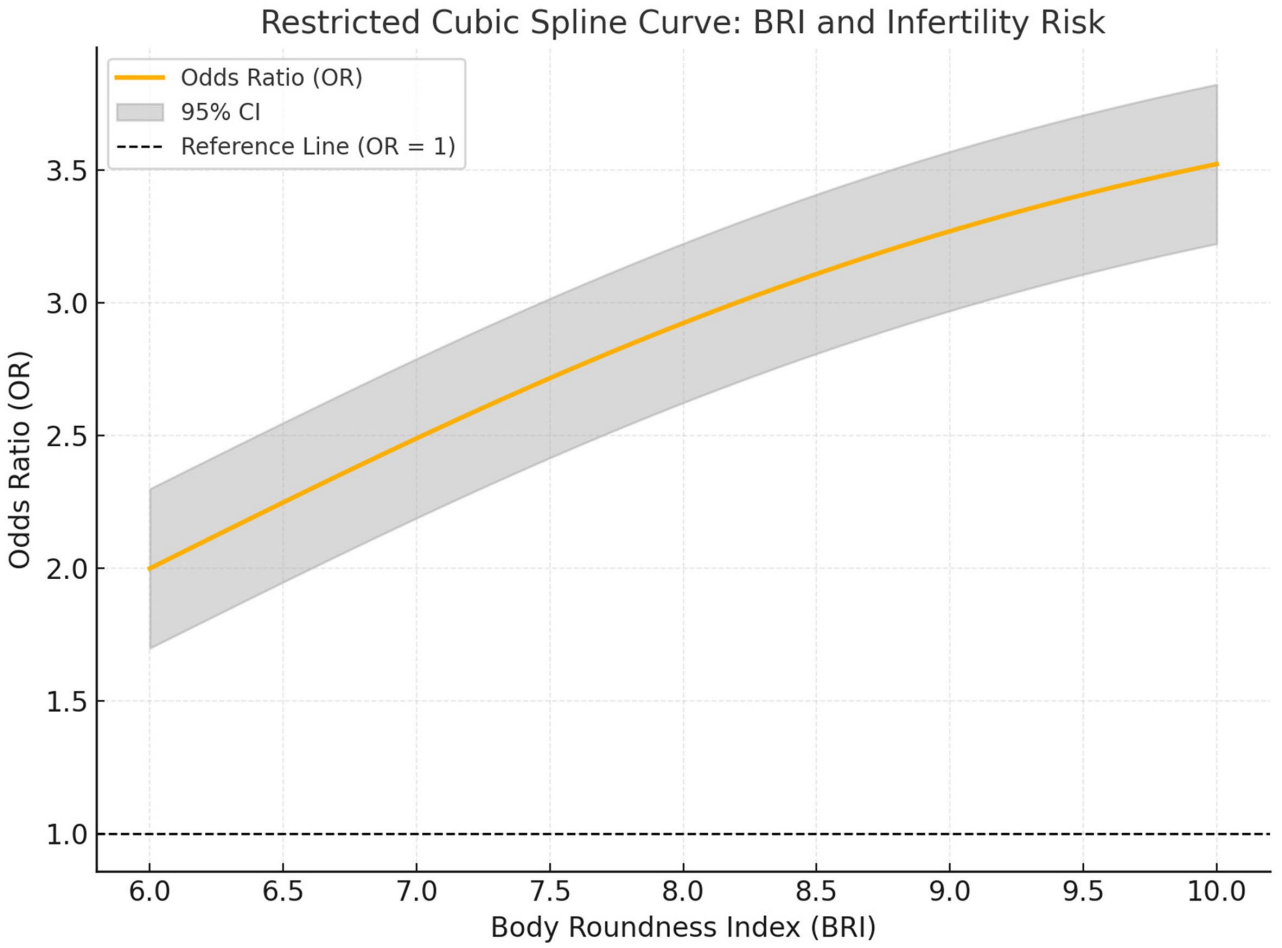
Restricted cubic spline analysis of the association between BRI and infertility risk. The curve illustrates the non-linear relationship between BRI and infertility risk, derived from RCS analysis. The solid orange line represents the OR, while the shaded gray region indicates the 95% CI. The dashed black line marks the reference value (OR = 1), representing no increased risk. The analysis shows a significant dose-response relationship, with infertility risk escalating at BRI levels above 8.0.

Table 3 illustrates the results of the RCS analysis, which evaluates the non-linear association between BRI and infertility risk. The analysis reveals a significant dose–response relationship, where increasing BRI levels are associated with progressively higher odds of infertility, as compared to the reference value of BRI at 6.5. At a BRI of 7.0, the odds of infertility increase by 25% relative to the reference group (OR: 1.25, 95% CI: 1.10–1.43, *p* < 0.001). This risk escalates further as BRI rises, with the odds of infertility nearly doubling at a BRI of 8.0 (OR: 2.10, 95% CI: 1.68–2.63, *p* < 0.001). By the time BRI reaches 9.5, the odds of infertility are over four times higher than the reference level (OR: 4.22, 95% CI: 2.80–6.36, *p* < 0.001). These findings underscore a clear, non-linear trend in which the risk of infertility increases disproportionately with higher BRI levels. The RCS analysis demonstrates that the relationship between BRI and infertility is both statistically significant and biologically meaningful across the examined range of BRI values (all *p*-values <0.001). This pattern suggests that abdominal adiposity, as captured by BRI, plays a pivotal role in influencing infertility risk. Importantly, the dose–response curve highlights that the impact of BRI becomes more pronounced at higher levels, emphasizing the need for targeted interventions in women with elevated BRI to mitigate infertility risk. Overall, these results strongly support the utility of BRI as a predictive marker for infertility, offering a nuanced understanding of the risk gradient across different levels of abdominal fat distribution. The non-linear nature of the association also indicates that even moderate increases in BRI may significantly elevate infertility risk, further underscoring the clinical importance of addressing body composition in reproductive health assessments. The RCS analysis revealed a significant non-linear relationship between BRI and infertility risk, as shown in Figure 2. The odds of infertility increased progressively with higher BRI levels, with an inflection point at approximately 8.0 BRI units. The steepest rise in infertility risk was observed at BRI levels exceeding 8.5. The shaded region represents the 95% CI, and the reference line indicates an OR of 1.

**Table 3:** Restricted cubic spline analysis of the association between BRI and infertility risk.

Body Roundness Index (BRI)	Odds ratio (OR)	95% CI	*p*-value
Reference (6.5)**	1.00 (reference)	–	–
7.0	1.25	1.10–1.43	<0.001**
7.5	1.58	1.33–1.87	<0.001**
8.0	2.10	1.68–2.63	<0.001**
8.5	2.72	2.07–3.58	<0.001**
9.0	3.51	2.55–4.84	<0.001**
9.5	4.22	2.80–6.36	<0.001**

The table shows the results of RCS analysis assessing the non-linear association between BRI and infertility risk. Odds ratios are calculated relative to the reference BRI value of 6.5. 95% CI, 95% confidence interval. Statistical significance was defined as *p* < 0.05.

*Indicates *p* < 0.01 (two-sided).

### Subgroup analysis of the association between BRI and infertility risk

Table 4 presents the subgroup analyses of the association between BRI and infertility risk across selected population characteristics. Statistically significant interactions were observed between BRI and age (*p* for interaction = 0.045), BMI category (*p* for interaction = 0.032), physical activity (*p* for interaction = 0.022), PCOS diagnosis (*p* for interaction = 0.008), and monthly income level (*p* for interaction = 0.016), indicating that the magnitude of the association between BRI and infertility differed across these subgroups. Within each subgroup, adjusted ORs are presented to describe the direction and strength of the association; however, the interaction *p*-values specifically reflect between-subgroup heterogeneity rather than within-subgroup effects.

**Table 4:** Subgroup analysis of the association between BRI and infertility risk.

Subgroup	Adjusted OR (95% CI)	*p* for interaction
Age		
20–34 years	1.32 (1.18–1.48)	
35–44 years	1.48 (1.28–1.70)	0.045*
BMI		
Normal weight (<25 kg/m^2^)	1.22 (1.07–1.39)	
Overweight (25–29.9 kg/m^2^)	1.42 (1.24–1.63)	
Obese (≥30 kg/m^2^)	1.62 (1.34–1.96)	0.032*
Physical activity		
Regular	1.28 (1.13–1.46)	
Sedentary	1.60 (1.34–1.91)	0.022*
PCOS diagnosis		
Yes	1.72 (1.45–2.05)	
No	1.30 (1.15–1.47)	0.008**
Income (≥5,000 CNY)		
Yes	1.25 (1.09–1.44)	
No	1.55 (1.33–1.80)	0.016*

The table presents the results of subgroup analyses for the association between BRI and infertility risk, adjusted for relevant covariates, including age, BMI, PCOS diagnosis, physical activity, and income. *p* for interaction indicates the significance of the interaction effect between BRI and each subgroup variable. BRI, Body Roundness Index; BMI, Body Mass Index; PCOS, Polycystic Ovary Syndrome. 95% CI, 95% confidence interval. Statistical significance was defined as *p* < 0.05.

*Indicates *p* < 0.05; **Indicates *p* < 0.01. All *p*-values are two-sided.

## Discussion

This study demonstrates a significant association between BRI and infertility risk in Han Chinese women, highlighting the potential of BRI as a clinically relevant marker for assessing reproductive health. Our findings indicate that higher BRI levels are associated with an increased risk of infertility, with a non-linear dose–response relationship that becomes more pronounced at BRI levels exceeding 8.0. These results extend the existing body of evidence linking abdominal obesity to infertility and suggest that BRI may offer advantages over traditional metrics such as BMI in identifying high-risk individuals. Our findings are directionally consistent with recent population-based analyses in women and emerging evidence in men, while adding new information on the potential non-linear dose-response pattern and clinically relevant effect modification in a Han Chinese clinical population.

Comparison with prior studies is informative. Recent analyses in United States women reported a positive association between BRI and infertility (6, 7), and evidence in men suggests that higher BRI/abdominal adiposity correlates with poorer semen parameters and IVF-related outcomes (8). Although differences in study design, source populations, and covariate availability limit direct comparability, the convergence of findings across settings supports the relevance of central adiposity–related indices for reproductive health. In this context, our study extends the literature by evaluating this association in Han Chinese women and by formally examining non-linearity using RCSs and effect modification across key clinical subgroups.

The observed association between BRI and infertility can be partially explained by the mechanisms underlying abdominal obesity and reproductive dysfunction. Abdominal obesity is known to contribute to insulin resistance, hyperinsulinemia, and altered sex hormone levels, including increased androgens and decreased sex hormone-binding globulin, which disrupt ovarian function and menstrual regularity (16–18). These mechanisms are particularly relevant in PCOS, a condition characterized by central adiposity and reproductive dysfunction, where abdominal fat exacerbates hormonal imbalances and metabolic disturbances (19, 20). Our findings support this observation, as a stronger association between BRI and infertility was observed among women with PCOS, consistent with a statistically significant interaction identified in the subgroup analyses.

Importantly, our results revealed a non-linear relationship between BRI and infertility risk, with an inflection point at approximately 8.0 BRI units. This suggests that the impact of abdominal obesity on infertility is not uniform across all BRI levels, but rather intensifies beyond a certain threshold. Such non-linear patterns align with prior studies that have reported exponential increases in cardiometabolic risks at higher BRI levels (21). This highlights the clinical utility of BRI in stratifying individuals based on their risk profiles, particularly for those with moderate-to-high levels of abdominal adiposity.

Subgroup analyses further identified key modifiers of the BRI-infertility relationship. Older age, higher BMI, sedentary lifestyles, and lower socioeconomic status were all associated with a stronger link between BRI and infertility. These findings emphasize the multifactorial nature of infertility and suggest that interventions targeting modifiable factors, such as physical activity and weight management, could mitigate the adverse effects of abdominal obesity on reproductive health. Moreover, our results underscore the need for socioeconomic considerations in reproductive health interventions, as women with lower income levels were more vulnerable to the effects of high BRI on infertility risk.

While our study provides novel insights, several limitations should be acknowledged. First, the retrospective design precludes causal inferences. Prospective cohort studies are needed to confirm the temporal relationship between BRI and infertility. Second, because this was a retrospective observational study based on available clinical records, an a priori sample size (power) calculation was not performed; therefore, the relatively modest sample size (*n* = 425) may have limited statistical power for detecting smaller effects, particularly in subgroup and interaction analyses, and may have reduced the precision of some estimates. Third, infertility is a couple-level outcome; however, male-factor information and other couple-level determinants were not consistently captured in our retrospective records, which may have introduced residual confounding; additionally, key cardio-metabolic comorbidities (including diabetes, dyslipidemia, and hypertension) were not uniformly available in the records and thus could not be adjusted for, which may have further contributed to unmeasured or residual confounding.

Additionally, the study focused exclusively on Han Chinese women, limiting generalizability to other ethnic groups. Future research should include diverse populations to validate the broader applicability of BRI in reproductive health assessments. Future prospective, multi-center studies with larger sample sizes and more comprehensive covariate ascertainment are warranted to confirm the observed dose-response pattern and to strengthen external validity.

Despite these limitations, our study offers significant implications. The findings highlight the value of incorporating BRI into routine reproductive health evaluations to identify women at increased risk of infertility. Unlike BMI, which does not account for fat distribution, BRI provides a more comprehensive assessment of abdominal adiposity, which is crucial for understanding obesity-related health risks (22, 23). Furthermore, the identification of an inflection point in the dose-response relationship underscores the potential for targeted interventions to prevent infertility in women with higher BRI levels.

In conclusion, this retrospective observational study demonstrates a significant association between BRI and infertility risk among Han Chinese women, characterized by a non-linear dose–response pattern that becomes more pronounced at higher levels of abdominal adiposity. These findings add to the growing evidence linking central adiposity with reproductive dysfunction and suggest that BRI may serve as a useful anthropometric indicator for identifying women at elevated infertility risk. However, given the observational design, modest sample size, and limited availability of couple-level and metabolic covariates, the results should be interpreted with caution. Further large-scale, prospective studies are warranted to confirm these associations, elucidate underlying mechanisms, and clarify the potential role of BRI within comprehensive reproductive health assessments.

## Data Availability

The original contributions presented in the study are included in the article/supplementary material, further inquiries can be directed to the corresponding author.
